# Erratum to: ‘Reference-free inference of tumor phylogenies from single-cell sequencing data’

**DOI:** 10.1186/s12864-016-2609-2

**Published:** 2016-05-10

**Authors:** Ayshwarya Subramanian, Russell Schwartz

**Affiliations:** Department of Biostatistics, Harvard T.H. Chan School of Public Health, 655 Huntington Street, 02115 Boston, USA; Department of Biological Sciences and the Computational Biology Department, Carnegie Mellon University, 5000 Forbes Avenue, 15213 Pittsburgh, USA

Unfortunately, the original version of this article [1] contained an error. Figures 2, 4 and 5 were incorrect and the captions for Figs. 4 and 5 were incorrect. Below are the correct figures and captions:

**Fig. 2:**
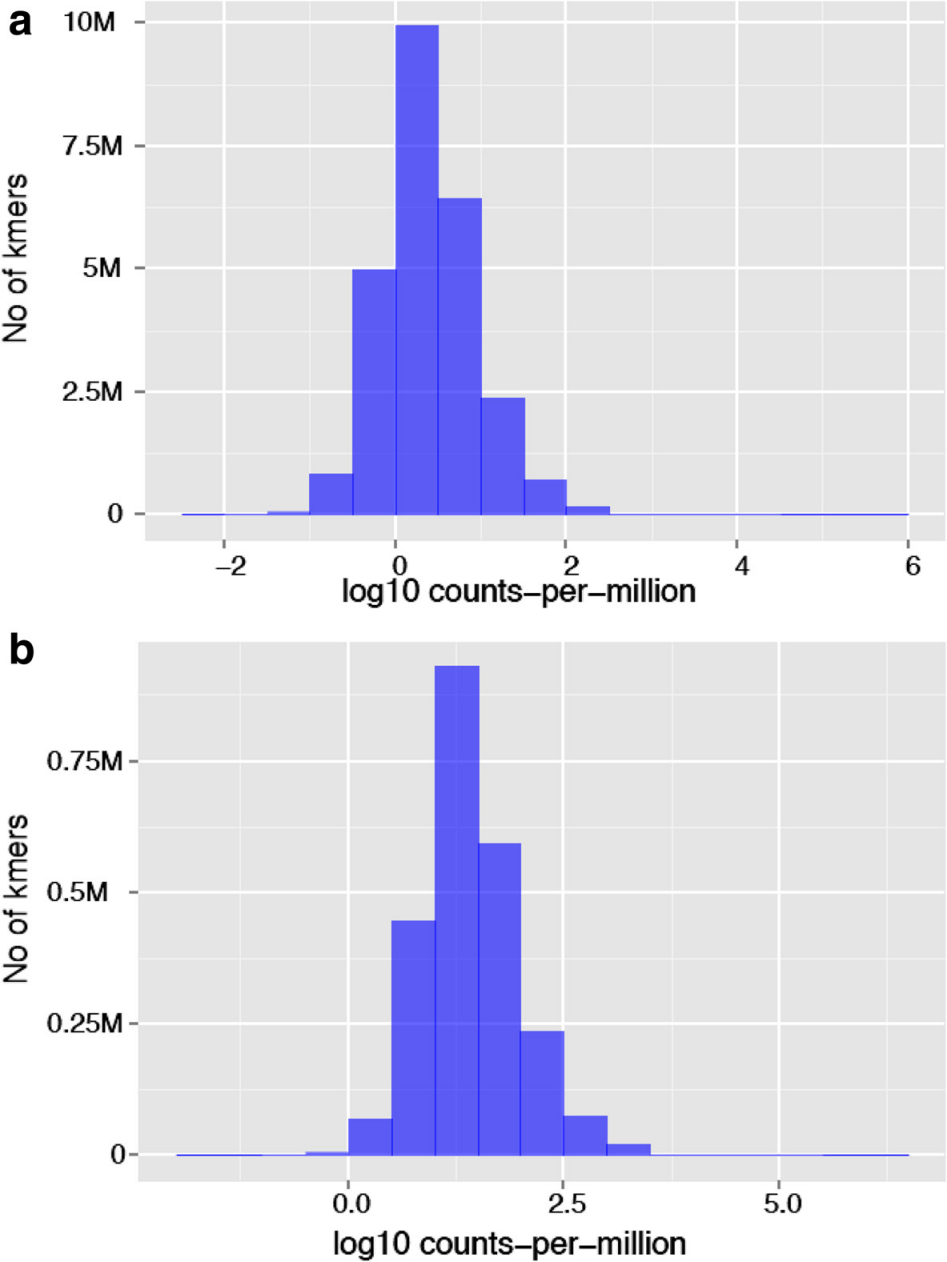
Histogram of k-mer relative abundances. Both 20- and 25-mer relative abundance densities appear log-laplacian. These data included 20- and 25-mers found in all tumor cells. **a** Histogram of 20-mer relative abundances in log10 scale. **b** Histogram of 25-mer relative abundances in log10 scale

**Fig. 4:**
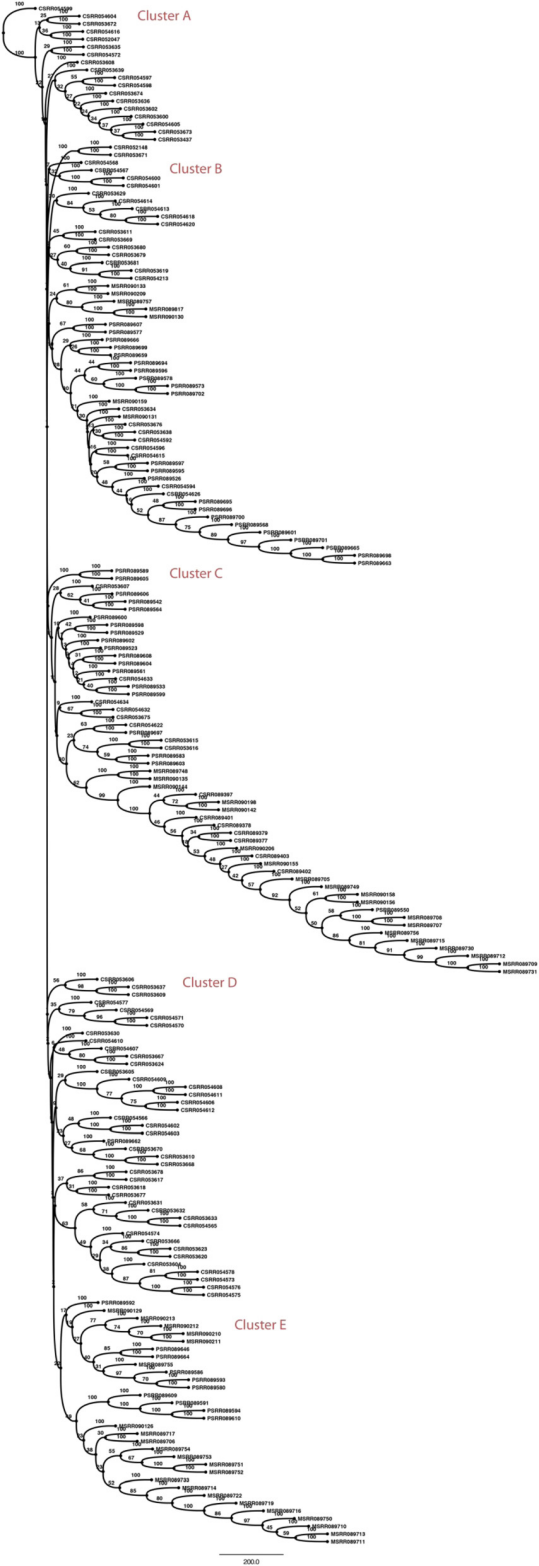
20-mer bootstrap consensus neighbor-joining tree built from T10 primary breast tumor cells (prefix C), T16 primary (prefix P) and metastatic data (prefix M). Distinct groupings of cells are labeled as clusters

**Fig. 5:**
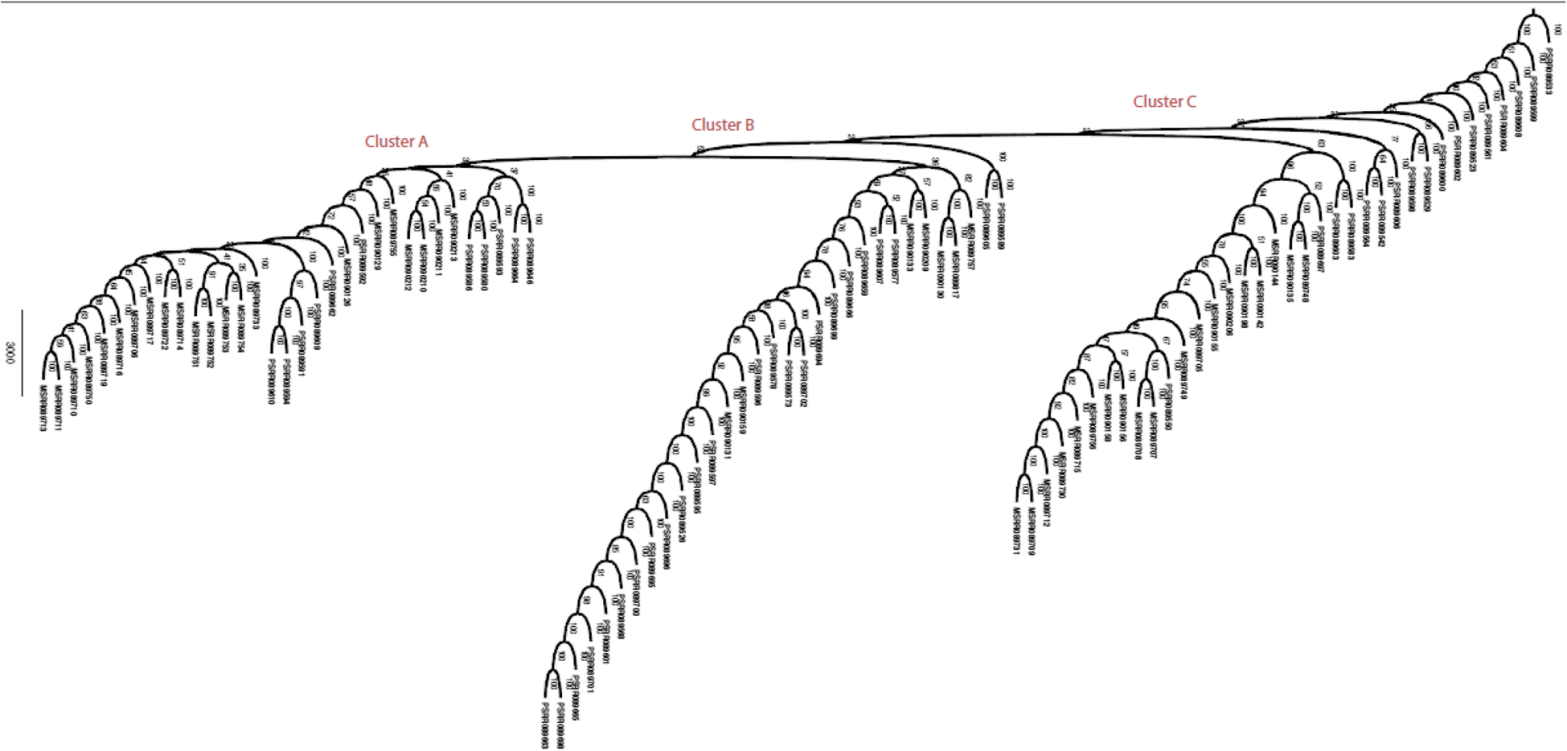
20-mer bootstrap consensus neighbor-joining tree built from T16 primary (prefix P) and metastatic data (prefix M). Distinct groupings of cells are labeled as clusters
